# Drug prices: is the sky the limit?

**DOI:** 10.1590/S1679-45082016ED3620

**Published:** 2016

**Affiliations:** 1 Hospital Israelita Albert Einstein, São Paulo, SP, Brazil

Drug prices are established mostly by the Big Pharma companies in America and Europe, and those prices are transmitted, when they manage to do so, to other countries. New drugs for hepatitis C, monoclonal antibodies used in cancer therapy or new inhibitors of kinases also used for cancer treatment, are examples of very expensive drugs. High prices are even higher in Brazil because of our taxing system, described many times by our press as crazy: in Brazil 34% of total drug price is tax. Imported drugs (the majority of new resources for cancer treatment are not available in our internal market) do not pay importation tax and IPI, if a medicine is imported in the name of an individual based on norms of our National Health Surveillance Agency (ANVISA). Imported drugs authorized by ANVISA and available in our country pay all taxes and in this case almost 60% of the prices are taxes. There are tributary waivers for a good number of drugs for some taxes, like PIS and COFINS: states taxes as ICMS do not have waivers.^(1)^

Some societies like the American College of Cardiology (ACC), Memorial Sloan Kettering Cancer Center (MSKCC) and the Institute for Clinical and Economic Review (ICER) try to evaluate what they call “the real value of a drug”. They elaborate tables comparing quality of life and survival years added when a drug is used, and check if the price of the drug correlates with those measures.^(2)^ A recent paper published in the journal Cancer by physicians of our North American sister, MD Anderson Hospital, analyzed treatments for hematological malignancies and calculated the incremental cost/effectiveness ratios by using US$ 50,000.00 per quality-adjusted life/year (QUALYS) as the breakpoint, which showed that tyrosine kinase inhibitors and monoclonal antibodies are over this mark. So, the costs of the majority of new treatments for hematological cancers are too high to be deemed cost/effective in the United States – this is their phrase entirely, and refers to United States' costs.^(3)^

Cancer treatment is getting more and more expensive and this trend does not show any evidence of abating, causing distress for patients.^(4)^ The national health system of Great Britain has an organ to decide which drugs the system will have and, consequently, which drugs patients shall receive. Very expensive monoclonal antibodies, for instance, are available in Great Britain, but they are not available in the public system, in which other medicines are offered without cost to the patients. The system includes drugs with definite favorable results, defined after randomized clinical trials. As Great Britain does not have a Constitution like ours, which promises all medical treatments for any clinical situation, there are no legal decisions ordering the state to give medicines for patients who need them. In fact, Great Britain manages very well without any Constitution since *Magna Carta*…

Big Pharma companies justify the new drug prices increase for paying research and developing of medicines. Drug market is not exactly an open market with lots of concurring actors; there are monopolies and duopolies on many drugs.^(5)^ Investing resources and research in new drug research is partly true, but not the whole history: most of new drugs are discovered and tested in phases 1 and 2 at small firms or universities, and Big Pharma gobbles the most promising of them. We do not know the real costs of drug development, as Big Pharma does not furnish their data, but it is possible to suppose that there is a sizable interest on those costs. One recent example of Big Pharma social conscience was Daraprim^®^. This old drug was about US$ 13,00 per pill and when one a Big Pharma bought the manufacture, the price changed for around US$ 700,00 per pill… in an old, venerable drug that has been in the market for years…^(5)^

In Brazil we have three serious problems involving drug therapy: prices here could be lower if taxes were withhold. Many new drug resources are not available because of bureaucratic norms, drug approval delays from Brazil's ANVISA, because some of the drug firms decide not to licensee their products in Brazil and because very expensive drugs can be prescribed by any physician. Our official ethical board insists that any physician should be free to prescribe whatever they think is the best for their patients. This leads to inadequate use: those drugs should be prescribed by physicians from university centers or by those who have recognized expertise in their use. There is also the possibility of importing drugs for patients from United States or Europe, or even from India, where medicines are cheaper, but this implies delays in treatments and expensive spends, as they have to be paid in dollars. The Brazilian Real is getting weaker and weaker against the dollar, which is a limitation. Judicial decisions are making our public health system spend a lot of resources to furnish free drugs for patients, and many of these patients are not the poor, but from middle class with information and enough money for paying good lawyers. They are within their rights, based on our Constitution, and if this document is not changed or at least improved, the public resources will be drained by those patients. As resources are finite, if they continue with this pace, others patients in Brazil will have count with less. And this does not sound fair.

Paraphrasing Lenin, who is still revered by some of our “Jurassic politicians”, what should be done? Change the Constitution for sure, less taxing for drugs and a coordinated international action to make Big Pharma companies to show their responsibility not only with actionists but also with humanity as a whole. If they have such a big expenses in developing new drugs, including the many drugs that never make to the market, nobody can make them produce without recovering costs: Big Pharma definitely are not philanthropic entities, and capitalism does not work that way. But we would like to know the real accounts and how they calculate their costs. Accounting at least in Brazil is known by its creativity, but if you have the actual data, you can interpret them. Show your data to the public, and then it will be possible to understand and perhaps improve the real price of essential drugs, including those of very restricted markets.
